# Metallic Nanoantioxidants as Potential Therapeutics for Type 2 Diabetes: A Hypothetical Background and Translational Perspectives

**DOI:** 10.1155/2018/3407375

**Published:** 2018-06-27

**Authors:** Oleh Lushchak, Alina Zayachkivska, Alexander Vaiserman

**Affiliations:** ^1^Department of Biochemistry and Biotechnology, Vasyl Stefanyk Precarpathian National University, 57 Shevchenko, Ivano-Frankivsk 76018, Ukraine; ^2^Laboratory of Epigenetics, Institute of Gerontology, 67 Vyshgorodska, Kyiv 04114, Ukraine

## Abstract

Hyperglycemia-induced overproduction of reactive oxygen species (ROS) is an important contributor to type 2 diabetes (T2D) pathogenesis. The conventional antioxidant therapy, however, proved to be ineffective for its treatment. This may likely be due to limited absorption profiles and low bioavailability of orally administered antioxidants. Therefore, novel antioxidant agents that may be delivered to specific target organs are actively developed now. Metallic nanoparticles (NPs), nanosized materials with a dimension of 1–100 nm, appear very promising for the treatment of T2D due to their tuned physicochemical properties and ability to modulate the level of oxidative stress. An excessive generation of ROS is considered to be the most common negative outcome related to the application of NPs. Several nanomaterials, however, were shown to exhibit enzyme-like antioxidant properties in animal models. Such NPs are commonly referred to as “nanoantioxidants.” Since NPs can provide specifically targeted or localized therapy, their use is a promising therapeutic option in addition to conventional therapy for T2D. NP-based therapies should certainly be used with caution given their potential toxicity and risk of adverse health outcomes. However, despite these challenges, NP-based therapeutic approaches have a great clinical potential and further translational studies are needed to confirm their safety and efficacy.

## 1. Introduction

Diabetes mellitus is one the leading causes of death in most countries across the globe. Over the last few decades, diabetes has emerged as an epidemic worldwide. Today 415 million people, about 9% of the adult population, have diabetes and this number is expected to increase to 642 million people during the next decade [1]. Type 2 diabetes (T2D), also called noninsulin-dependent or adult-onset diabetes, accounts for roughly 90% of all diabetes cases worldwide. The pathophysiology of this disease is characterized by peripheral insulin resistance, declining *β*-cell function, and impaired glucose metabolism in the liver [2]. The causes and manner of the development of T2D are associated with numerous factors, including genetic predisposition, age, unhealthy nutritional habits, decreased physical activity, and stressful life conditions. Lifestyle factors contributing to this disease (e.g., diet and exercise) may be controlled through lifestyle changes [3]. However, in some cases, impairments in insulin secretion and/or sensitivity are too severe to be corrected by lifestyle changes. In these cases, the use of oral hypoglycaemic drugs or even insulin injections is the next treatment option to control blood sugar levels [4].

## 2. Treatment of T2D: Old Problems and New Solutions

T2D patients usually receive oral or injectable medications to improve the production and function of insulin. However, antidiabetic therapies are still far from perfect. Indeed, currently used antidiabetic medicines, which act primarily by suppressing hepatic gluconeogenesis and improving insulin sensitivity, have many side effects. For example, oral hypoglycemics including the first-line antidiabetic drug, metformin, often cause gastric distress leading to nausea and diarrhea [4]. Moreover, oral drugs may stop working for T2D patients and they may ultimately require insulin therapy to achieve and maintain adequate glycemic control. In addition, even though considerable technological advances have been made over the past years in the treatment of T2D, it remains difficult to maintain proper glucose levels using conventional treatment options. Indeed, about half of diabetic patients fail to achieve target blood glucose levels when using common therapeutic modalities [5]. Therefore, many attempts have been made in recent years to develop alternative treatment options for management of T2D [6].

The use of natural health products is one of the most promising tools in T2D management. Among them, there are several trace metals, such as chromium, selenium, vanadium, molybdenum, and magnesium, which are known to exert hypoglycemic activity possibly due to their insulin-mimetic effects [7–10]. For example, chromium has shown the benefit in the treatment of T2D, particularly in conditions of chromium deficiency or when diabetes is poorly controlled [11]. From these findings, it can be assumed that dietary supplementation with well-studied trace metals can be a promising treatment option for T2D patients, complementary to approved pharmacological therapies. In this context, the use of metal nanoparticles (NPs) seems highly attractive for biomedical applications. NPs, nanosized materials with a dimension of 1–100 nm, exhibit unique size-related optical, electronic, and catalytic properties that differ significantly from those observed in the corresponding bulk materials due to their high surface area and nanoscale size [12, 13]. One important advantage of artificially engineered nanomaterials is that they may be well controlled for appropriate usage due to their tuned physicochemical properties; it provides an opportunity to directly influence interactions between nanomaterials and cells [14]. Finally, with respect to the biomedical applicability of NPs, a crucial point is that the interaction of NPs with proteins may influence both protein structure and function providing a means to influence the enzyme response to disease states [12, 15].

Nanotechnology-based systems provide apparent benefits in terms of increased bioavailability, decreased dosing frequency, prevention from degradation in the harsh gastric environment, high site specificity and minimal side effects [6]. NP drug delivery systems have attracted considerable attention due to their ability to overcome multiple biological barriers and release a therapeutic load in the optimal dose range [16]. Currently, numerous NP formulations including liposomes, nanostructures, polymer and metallic NPs, stimuli-responsive NPs, and nanofabricated devices are extensively used to deliver both small-molecule therapeutic agents and different classes of biomacromolecules, such as proteins, DNA, and RNA. These formulations are also used to diagnose and monitor the onset and progression of diseases [17]. Over the past two decades, clinical implementation of nanotechnology has led to both diagnostic and therapeutic advances in treatment of various chronic pathologies, including cardiovascular diseases [18], neurodegenerative disorders including Alzheimer's and Parkinson's diseases [19], and cancer [20], as well as diabetes [21, 22].

Currently, naked (not loaded with therapeutics) metallic NPs are increasingly used in a variety of biomedical applications [23, 24]. Metallic NPs may be synthesized and/or modified with diverse surface functionalities, thereby allowing them to be conjugated with different ligands, antibodies, vesicles, and drugs therefore increasing their potential clinical utility [25]. In this opinion paper, we focus on the potential therapeutic applications of metallic NPs in T2D management.

## 3. Role of Reactive Oxygen Species in Pathogenesis of T2D

Oxidative stress levels and associated chronic inflammation are known to be significantly increased in patients with metabolic syndrome and T2D [26, 27]. A potential means of using NPs for therapeutic purposes is related to their ability to modulate the level of oxidative stress, thus making them especially important in the context of the topic under consideration [28]. Free radicals such as reactive oxygen species (ROS), which are generated in the mitochondria during normal metabolic processes, are important second messengers that support signal transduction pathways implicated in normal cell functions including survival, proliferation, differentiation, and apoptosis [29]. Under normal physiological conditions, cellular ROS levels are strongly controlled by specific antioxidant enzymes, including superoxide dismutase (SOD), catalase (CAT), and glutathione peroxidase (GPX) and by exogenous antioxidants such as flavonoids, vitamin E, ascorbic acids, and glutathione (GSH) [30]. Under pathological conditions such as T2D, abnormally large ROS concentrations may damage a wide variety of biomolecules including lipids, proteins, and nucleic acids. ROS can also result in permanently disturbed patterns of gene expression and signal transduction [31]. Chronic extracellular hyperglycemia causes elevated production of ROS by the mitochondrial electron-transport chain and thus leads to disturbed cell redox state and abnormal expression of genes of insulin sensitivity [32]. Oxidative stress thereby plays a crucial role in hyperglycemia-triggered tissue damage and is regarded as one of the key initial events in T2D onset and progression. Chronic hyperglycemia also results in an increase of the generation of advanced glycation end products (AGEs), a group of modified lipids and/or proteins with damaging potential [33]. Overproduction of AGEs leads to an enhanced ROS formation and impaired antioxidant defense which results in a detrimental cycle since the generation of AGEs can be induced under oxidative conditions [33]. Therefore, AGEs are thought to play a significant role in the pathogenesis of T2D. Another important contributing factor in pathogenesis of T2D is accelerated telomere shortening. Telomeres are nucleotide repeated sequences that cap the chromosome ends and shorten with every cell division in absence of the telomerase activity. Age-associated telomere attrition, however, depends not only on the replicative shortening but also on the level of oxidative stress (known to be, in turn, contributing to most age-related chronic diseases), because of a deficiency in the repair of telomere-specific damage [34]. Shortened telomere lengths were revealed in pancreatic beta cells in T2D patients, thus potentially resulting in an impaired capability for proliferation, secretion of insulin, and in accelerated cell death [35]. There is evidence from animal models that insulin resistance can be induced through telomere attrition in adipose tissue as well. Telomere attrition depends on the level of oxidative stress [34]. Taken together, currently available data suggest that hyperglycemia, oxidative stress, and telomere attrition in both pancreatic beta cells and adipocytes may cumulatively form a vicious cycle contributing to the pathogenesis of T2D. Therefore, modulating these processes by innovative nanotechnology-based therapeutics could provide a promising approach for the prevention and progression of T2D and its complications.

In the last few decades, much hope was placed on the prevention and therapy of T2D with antioxidants owing to their ability to counteract oxidative stress by scavenging ROS. Oxidative stress occurs when ROS production exceeds the endogenous antioxidant defense capacity. Therefore, given that T2D-associated conditions are generally accompanied by a higher oxidative stress level, it was believed that patients suffering from this disease would benefit from exogenous antioxidant supplementation as adjunct therapy [36]. However, clinical antioxidant trials have been largely ineffective so far in preventing and managing T2D. Most of them gave inconclusive or even negative results [31, 37, 38]. Although there is robust experimental evidence for beneficial outcomes of the dietary antioxidant consumption, most interventional studies have failed to demonstrate any health benefits of antioxidants. This contradiction is commonly referred to as the “antioxidant paradox” [37, 39]. One possible explanation for this is that oxidative stress and inflammation accompanying T2D are closely linked to pathophysiological conditions which can mutually induce each other. From this, it can be assumed that the failure of clinical antioxidant trials could result from an inability to develop medications that specifically target both oxidative stress and inflammation or from failure to apply both anti-inflammatory agents and antioxidants simultaneously [39]. Moreover, it might result from the use of substances that block several inflammatory and/or prooxidative pathways but strengthen others. In addition, most orally administered antioxidants have limited absorption profiles and consequently have low bioavailability resulting in insufficient concentrations at the target site [40, 41]. Therefore, novel agents with antioxidant capabilities that have improved bioavailability and that may be delivered to specific target tissues have been actively developed in recent years.

## 4. Metallic NPs as Potential Modulators of T2D-Induced Oxidative Stress

Metallic NPs such as magnetic, silver, and gold NPs seem to have promising potential for use in prevention and treatment of disorders caused by excessive generation of ROS [25]. NPs, among other nanomaterials, are increasingly used for various biomedical applications owing to their exceptional and tunable biophysical properties dependent on their size and shape [22, 42]. Indeed, NPs differ substantially from their bulk analogs since their surface areas are significantly greater and contain a larger fraction of atoms [43]. Moreover, the surface-to-volume ratio is inversely related to particle size. Smaller NPs have larger ratio; therefore, the number of reactive sites on the surface of a NP is regulated by the particle size [44].

The NP-induced effects are mediated by their enhanced catalytic activity, which is likely related to the high ratio of electrons remaining on the particle's surface, increasing the ability of the NP to transform the substrate. Smaller NPs have higher catalytic activity than larger NPs owing to their greater surface area [45]. In addition, chemical reactivity substantially increases with decreasing particle size [43]. High chemical reactivity of NPs is commonly attributed to dangling bonds (unsatisfied valences on immobilized atoms located on the surface of NP) which consequently make their surface unstable and highly reactive [46]. Due to these unique properties of NPs, their clinical implementation would likely provide many benefits compared to conventional treatment modalities with drugs which have multiple side effects because of their insufficient and off-target activity.

### 4.1. NP-Induced ROS Generation

The most common negative outcome related to the therapeutic application of NPs is the excessive generation of ROS which is regarded as a key factor in NP-induced toxicity [25, 47, 48]. Both large surface areas and reactive surfaces contribute to the oxidizing capacities of the NPs. The mitochondrion is an organelle crucially involved in the NP-induced generation of cellular ROS due to the capacity of NPs to depolarize mitochondrial membrane potential and to interfere with the electron-transport chain via the activation of nicotinamide adenine dinucleotide phosphate- (NADPH-) related enzymes [49]. NP exposure can thus lead to blocking the mitochondrial electron-transport chain, consequently increasing the cellular levels of superoxide radicals through electron transfer from respiratory carriers to molecular oxygen. Other mechanisms involved in the generation of intracellular ROS by NPs include the catalysis of free-radical reactions, the interaction of NPs with different cellular components of redox active proteins (e.g., NADPH oxidase), as well as the interplay with cellular surface receptors and activation of various intracellular signaling pathways [49]. The NP-induced toxic state can cause elevated expression of proinflammatory cytokines and activation of inflammatory cells, including neutrophils and macrophages, which can result in an increased generation of ROS. The potential of different metallic NPs for generating ROS depends on their chemical composition, particle size, surface area, and shape [25], as well as on the mode of interaction with cells, aggregation, inflammation, and pH of the medium [50]. Importantly, on the cellular level, the amount of ROS generated is dependent on the concentration of NPs to which the cell is exposed. Exposure to low concentrations results in an improvement of the endogenous antioxidant defense system to combat the damaging consequences of oxidative stress and recover the redox balance, while exposure to high NP concentrations provokes excess ROS formation, overwhelming antioxidant systems and causing cytotoxicity and inflammation [51]. Determination of maximal effective doses therefore is critical to avoid negative outcomes.

### 4.2. Nanoantioxidants

Recently, some nanomaterials, including metallic NPs, were unexpectedly shown to exhibit enzyme-like antioxidant properties by being able to scavenge free radicals and decrease ROS concentrations [52, 53]. Such NPs are commonly referred to as “nanoantioxidants.” Nanoantioxidants include both NPs functionalized with antioxidants or antioxidant enzymes for functioning as an antioxidant delivery system and nonorganic NPs with intrinsic antioxidant properties. Significant antioxidant properties such as SOD-, CAT-, oxidase-, and peroxidase-mimicking activities have been demonstrated for metallic NPs produced from cerium oxide (nanoceria) [54–56], iron oxide [57, 58], cobalt oxide [59], copper oxide [60], manganese dioxide [61], and vanadium pentoxide [62], as well as from noble metals such as gold [63, 64], silver [65], and platinum [66].

**Figure 1:**
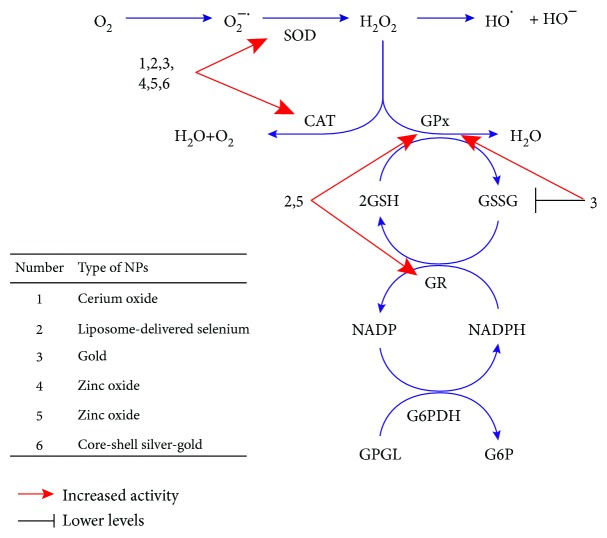
NPs can prevent diabetes-induced oxidative stress by affecting different steps of free-radical metabolism. Many NPs including all of those presented in Table 1 are able to restore the activities of superoxide dismutase (SOD) and catalase (CAT) that are often decreased under diabetic conditions. Lower oxidative damages to cellular macromolecules are achieved by decreasing the levels of superoxide anion (O_2_^−^) or preventing the generation of hydroxyl radical from hydrogen peroxide (H_2_O_2_). Additionally, t*he liposome-delivered selenium and zinc oxide* NPs can affect the activities of glutathione peroxidase (GPx) and glutathione reductase (GR), thereby increasing the H_2_O_2_ detoxification by glutathione-dependent system. Decreased concentration of oxidized glutathione (GSH) and increased activity of GPx are also observed under treatment with the gold NPs.

The precise molecular mechanisms determining the antioxidant capabilities of metallic NPs remain largely unclear. The antioxidant capabilities of these NPs can likely be attributed to their high surface-to-volume ratio, electronic configuration, catalytic and redox properties, and oxygen vacancy defects [52, 53]. The antioxidative behavior of metallic NMs may to a large extent depend on their capacity to oscillate between different multioxidation states. For example, antioxidant properties of nanoceria NPs, which are considered to be one of the most promising nanomaterials due to their catalytic properties, are thought to be related to the presence of oxygen vacancies on their surfaces and also to the autoregenerative cycle of their two oxidation states, Ce^3+^ and Ce^4+^ [67]. Indeed, as shown by X-ray absorption near edge spectroscopy and X-ray photoelectron spectroscopy, the concentration of Ce^3+^ relative to Ce^4+^ can be substantially increased as the particle size decreases, and such loss of oxygen due to reduction of Ce^4+^ to Ce^3+^ is accompanied by the formation of an oxygen vacancy on the surface of the NP [52, 67]. Such ability of this NP to oscillate among these multioxidation states can definitely contribute to its ROS-scavenging ability. Moreover, there is persuasive evidence that NPs including, for example, CeO_2_ NPs, can modulate key antioxidant pathways such as Nrf2 [53]. Furthermore, interactions of NPs with cellular macromolecules including proteins, lipids, and nucleic acids could be of great importance for these processes. A wide variety of sites of the NP protein interaction is expected through the diversity of protein structure, and the kinetics of NP protein interaction can significantly depend on the NP structure, as well as protein availability and duration of interaction [53]. Due to these properties, NPs can largely affect the cellular redox environment by either stimulating or inhibiting ROS generation under certain conditions. In addition, the hormetic phenomenon referred to as a “biphasic dose-response relationship characterized by a low-dose stimulation and high-dose inhibition” may likely play an important role in redox-modulating effects of NPs [68]. Under the conditions of oxidative stress, particular NPs could likely prevent key biomolecules from oxidative damage, thereby causing health benefits and disease prevention. The hypothetical mechanisms through which NPs can prevent diabetes-induced oxidative stress are presented in Figure 1.

The capability of metallic NPs to scavenge free radicals was repeatedly shown in *in vitro* studies [69–71], as well as in animal models such as *Drosophila melanogaster* [72, 73], mice [74], and rats [75–78]. Most compelling evidence for the ability of metallic NPs to protect against diabetes-related oxidative stress was obtained in the streptozotocin-induced diabetic rat model. Streptozotocin is known to destroy insulin-producing pancreatic beta cells, thereby leading to hypoinsulinemia and hyperglycemia in exposed animals. Since in this model hyperglycemia arises owing to the hypoinsulinemia in the absence of peripheral insulin resistance, it most closely mimics type 1 diabetes but can also be used for induction of T2D under certain conditions [79]. A summary of the main findings from these studies is provided in Table 1. As we can see from the table, administration of metallic NPs, independently on the route of administration, in most cases led to an improvement of metabolic indices and protection from diabetes-induced oxidative stress in streptozotocin-treated rats. These findings demonstrate that catalytic NPs could represent a promising therapeutic approach for patients with pathological conditions related to elevated levels of oxidative stress, including T2D.

## 5. Conclusion

The use of metallic NPs in clinical practice has many advantages, including their superior biocompatibility and stability, low operational and capital expenses, and reduced environmental impacts [87]. The development of metallic NPs with antioxidant properties seems a particularly promising therapeutic option, since it might provide specifically targeted or localized therapy [88]. The unique opportunities from the clinical application of nanoantioxidants are related to the fact that they may be made larger than the cutoff size for kidney filtration (~10 nm), thereby prolonging the circulation period in comparison with small molecules [28]. They may also be further designed to avoid rapid clearance by phagocytes or to target specific sites and organs. This allows them to be used in smaller but more effective doses, thus minimizing potential adverse impacts. Due to these properties, treatment with nanoantioxidants might represent a promising therapeutic option, in addition to conventional therapy, in patients with T2D. Finally, NP-based therapeutic approaches have a great clinical potential and certainly further translational studies are needed to confirm their safety and efficacy and overcome the known risks of toxicity and potential for adverse health outcomes at higher doses.

## Figures and Tables

**Table 1:** An overview of NP-induced metabolic outcomes and markers of oxidative status in streptozotocin-induced diabetic rats.

NP formulation	NP dose; route of administration	Metabolic outcomes	Markers of oxidative status	Ref.
Cerium oxide NPs	60 mg/kg per day for 2 wk; intraperitoneal injection	Increase of high density lipoprotein level; decrease of adenosine diphosphate/adenosine triphosphate (ADP/ATP) ratio, cholesterol, triglyceride, and low-density lipoprotein levels	Recovery in normal antioxidant enzyme activity and oxidative stress level	[80]
Cerium oxide NPs	65 or 85 mg/kg; intraperitoneal injection	Recovery in body weight, total thiol molecules, lipid peroxidation levels, and ADP/ATP ratio	Recovery in antioxidant enzyme activity	[81]
Liposome-delivered selenium NPs	0.1 mg/kg per day for 21 days; oral administration	Recovery in serum glucose and insulin and pancreatic malondialdehyde, nitric oxide, tumor necrosis factor-α, and prostaglandin F2α levels; improvement in immunohistochemical indices (insulin and glucagon)	Recovery in pancreatic SOD, CAT, glutathione, glutathione peroxidase, and glutathione reductase levels	[82]
Gold NPs	2.5 mg/kg for 7 days; intraperitoneal injection	Improved lipid profile and kidney functions; no evidence of separation of nuclear membrane in euchromatic nuclei of beta cells	Increased SOD, CAT, and glutathione peroxidase activities; lowered oxidized glutathione levels	[83]
Core-shell silver-gold NPs	0.5 or 1 ml per day for 21 days; oral administration by gastric intubation	Restoring the normal glucose and serum insulin levels and glucokinase activity; reducing the lipid profile; anti-inflammatory effect assessed using inflammatory markers IL-α and C-reactive protein; decreased level of necrosis of hepatocytes	Suppressing the oxidative stress and elevating the antioxidant defense system	[84]
Zinc oxide NPs	1, 3, and 10 mg/kg per day for 56 days; oral administration by gavage	Improved glucose disposal, insulin levels, and zinc status compared to rats supplemented with zinc sulfate	Altered activities of erythrocyte antioxidant enzymes, raised levels of lipid peroxidation, and a marked reduction of total antioxidant capacity in rats administered with high dose of NPs	[85]
Zinc oxide NPs	10 mg/kg per day for 30days; oral administration	Increased sperm count and motility	Increased activity of SOD, CAT, glutathione peroxidase, glutathione reductase, and glutathione-S-transferase; decreased malondialdehyde and increased glutathione levels in testicular tissue	[86]

## Abbreviations

AGEs: Advanced glycation end products
GSH: Glutathione
GPx: Glutathione peroxidase
GR: Glutathione reductase
GSSG: Oxidized glutathione
G6P: Glucose-6-phosphate
G6PDH: Glucose-6-phosphate dehydrogenase
CAT: Catalase
NP: Nanoparticle
NADPH: Nicotinamide adenine dinucleotide phosphate
ROS: Reactive oxygen species
SOD: Superoxide dismutase
T2D: Type 2 diabetes
6PGL: 6-phosphogluconate.
